# Platelet-Rich Fibrin: An Autologous Fibrin Matrix in Surgical Procedures: A Case Report and Review of Literature

**Published:** 2012

**Authors:** Majid Eshghpour, Mohamad Reza Majidi, Amir Hossein Nejat

**Affiliations:** 1 *Department Of maxillofacial surgery,Maxillofacial research committee, Mashhad Dental faculty, Mashhad, Iran*; 2 *Ear, Nose, Throat, Head and Neck surgery Research Center, Mashhad University of Medical Sciences, Mashhad, Iran*; 3 *Member of Student Research Committee, Mashhad dental faculty, Mashhad, Iran*

**Keywords:** Blood sample, Fibrin matrix, Healing, Platelet-rich fibrin, Surgery

## Abstract

**Introduction:**

The healing process after surgery is a challenging issue for surgeons. Various materials and techniques have been developed to facilitate this process and reduce its period. Fibrin adhesives are often used in cardiothoracic and vascular surgery to seal diffuse microvascular bleeding and in general and plastic surgery to seal wound borders. This Case report and literature review will introduce the various usages of platelet-rich fibrin in different surgical procedures and the method of producing the matrix.

**Case Report:**

A 24-year old man with periorbital skin avulsion treated with PRF membrane has been reported and discussed in this paper.

**Conclusion:**

Platelet-rich fibrin is a natural autologous fibrin matrix, which can be produced with a simple blood sample and a table centrifuge. The material has been used in a wide range of surgical procedures to shorten the healing period and reduce post-surgical complications.

## Introduction

The healing process following surgical procedures is a challenging issue for surgeons. A wide range of materials and techniques have been proposed to enhance healing and reduce the time required to complete the process. In the past 30 years fibrin adhesives have been used in many protocols (1, 2). Platelet-rich fibrin (PRF) was first introduced in France by Choukroun and colleagues (5), and has been most widely used in cardiothoracic surgery (2), vascular surgery (2), general surgery (3), plastic surgery (3), to reduce postoperative hematoma (4), and in sinus lift procedures and implantation (8). PRF has been effective in plastic surgeries of the face and is an excellent choice for such surgeries (9). In this paper we describe a skin avulsion that was treated with PRF membrane.

## Case Report

**Fig 1:**
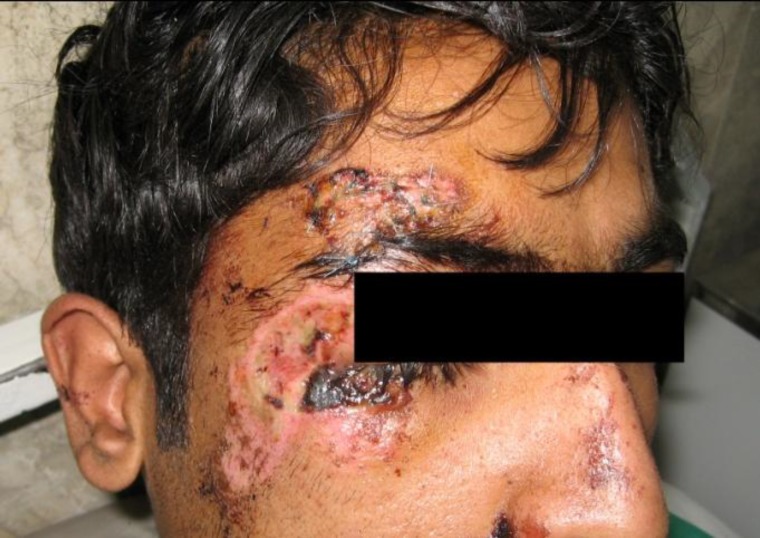
The avulsion area before treatment

A 24-year old man who suffered a motorcycle accident was referred to Shahid Kamyab Hospital. An examination of the subject revealed an avulsion of his periorbital skin that was 2 × 3 cm in size (Figure 1). The patient had no medical history.

**Fig 2:**
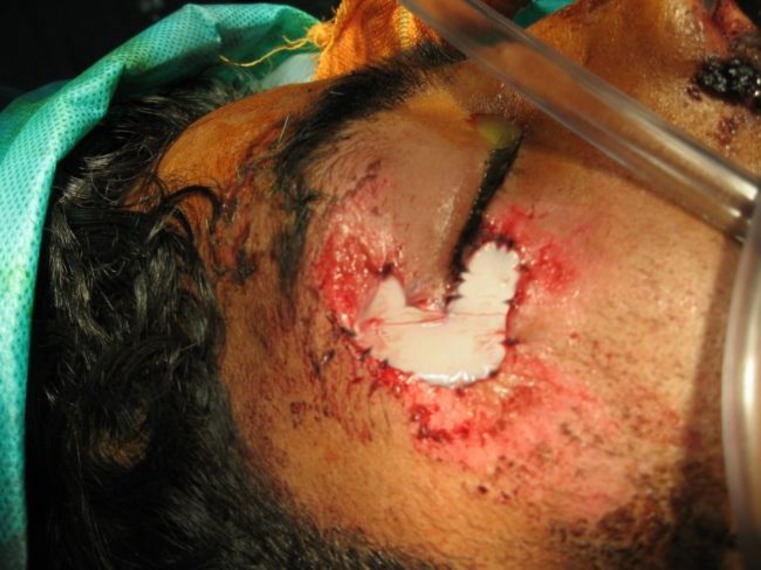
The PRF membrane sutured in place with 5-0 Nylon suture

To reconstruct the avulsed area a PRF membrane was made using 10 cc of blood obtained from the brachial vein. PRF was obtained following centrifugation of the blood sample. The membrane was then made and placed on the area of avulsion to reduce the amount of scar tissue following the healing process. The membrane was sutured in place with 5-0 Nylon suture material (Figure 2). 

**Fig 3:**
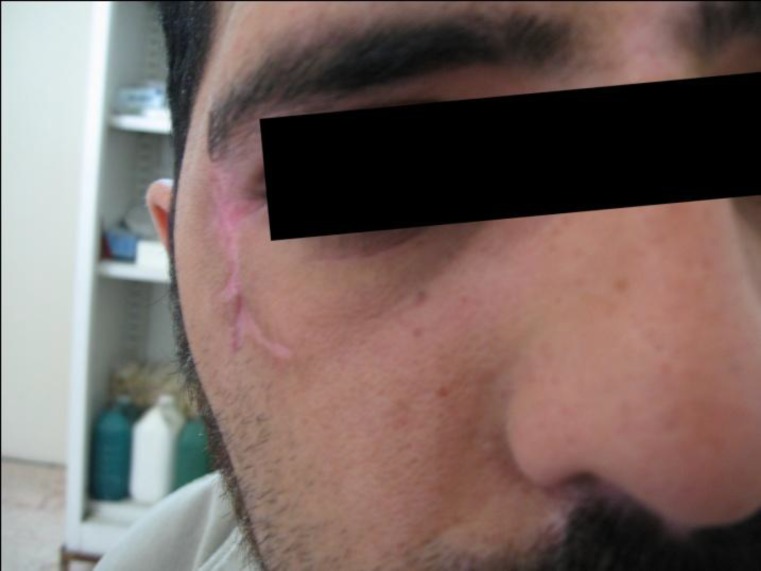
Healing site after 8 weeks

After 8 weeks the subject demonstrated uneven healing of the area with a small amount of scar tissue (Figure 3).

## Discussion

One of the great challenges that faces a surgeon after each intervention is the complexities and problems of the healing process. The use of fibrin adhesives in many protocols has been well documented over the past 30 years (1,2). Fibrin adhesives are often used in cardiothoracic and vascular surgery, and are used to seal diffuse microvascular bleeding through spray application (2). Above all, fibrin adhesives are well known for their use in the sealing of wound borders and facilitating cutaneous reuse in general and plastic surgery (3). In addition to the mechanical properties of the adhesive, the biological properties of fibrin are used to promote cicatrization. Beside the capacity to accelerate healing, sealing with fibrin adhesive is conventionally used for reducing postoperative hematoma (4).

*Production Procedure*

**Fig 4:**
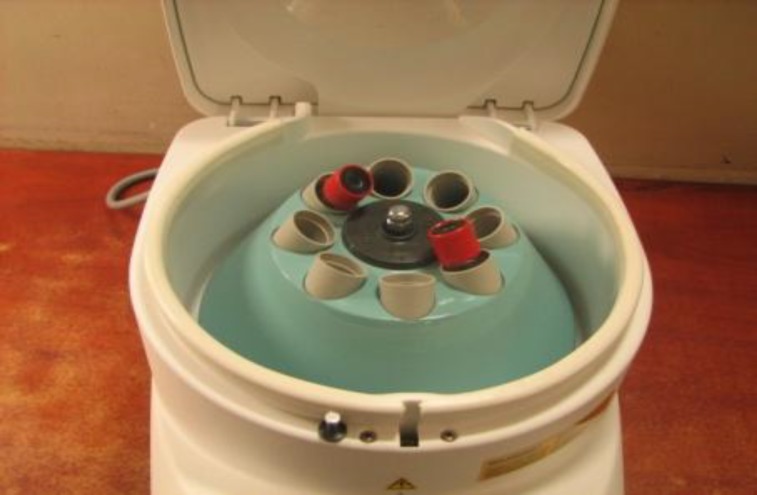
Table centrifuge

PRF is a natural fibrin matrix which was first developed in France by Choukroun and colleagues (5). The technique of producing PRF is very simple and needs nothing more than a blood sample and a PC-02 table centrifuge (Figure 4). 

**Fig 5:**
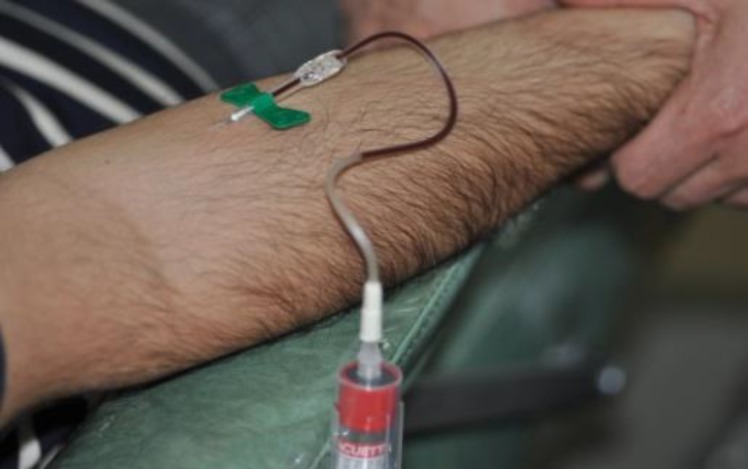
Taking a blood sample

**Fig 6:**
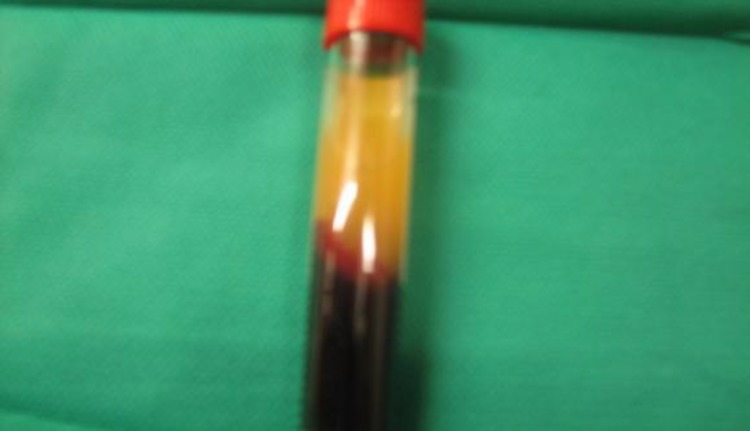
The 3 layers in a centrifuged blood sample: top, plasma; middle, PRF; bottom, red corpuscles

**Fig 7:**
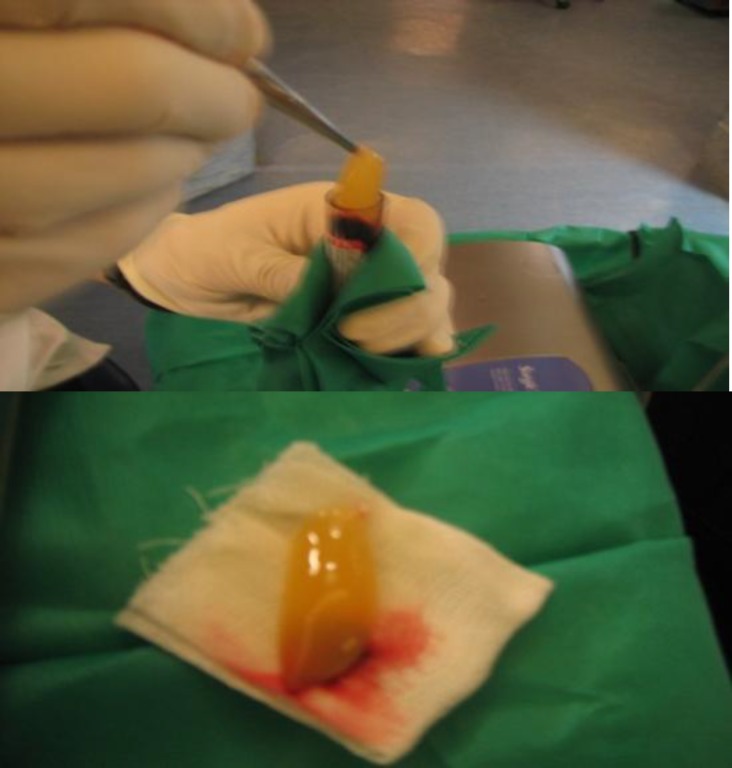
Following PRF extraction the clot of red blood cells attached to the bottom of the PRF matrix needs to be cut off

The protocol is as follows: A blood sample is taken (Figure 5) without any anticoagulant in 10-mL tubes, which are immediately centrifuged at 3000 rpm for 10 minutes. Tubes should be paired in the device (with an odd number of samples, the unpaired one could be paired with a water-filled tube). Following centrifugation, three parts are seen at the end in the tube (Figure 3): red corpuscles at the bottom, a fibrin clot representing PRF matrix in the middle (Figure 6), and acellular plasma (PPP) at the top. By extracting the matrix from the tube with forceps and cutting off the red blood corpuscles the PRF matrix (PRFM) can be obtained (Figure 7). Note that the success of this technique entirely depends on the speed of blood collection and transfer to the centrifuge as no anticoagulant substance is used. To produce a fibrin membrane, fluid trapped in the fibrin matrix is driven out and a very resistant autologous fibrin membrane is obtained (6).

*Uses of PRF*

*1. Soft Tissue Repair*

Visser and colleagues (2011) tried to enhance the healing process of defects of the patellar tendon (PT) in dogs. They created a defect in the central third of the PT in both hind limbs of each dog. An autologous PRF membrane was implanted in 1 defect per dog and the contralateral defect was left untreated. Dogs (n= 4 per time period) were euthanized at 4 and 8 weeks after surgery. Both treated and control defects were filled with repair tissue by 4 weeks. No significant difference in the histologic quality of the repair tissue between the control and PRF membrane-treated defects was observed at either time point. However, at both time points the cross-sectional area of the PRF membrane-treated tendons was significantly greater (at least 2.5-fold as great). The results of the study indicated that the use of PRF membrane did not enhance the rate or quality of tendon healing in PT defects. However, it did increase the amount of repair tissue within and surrounding the defect (7).

*2. Osteointegration*

Inchingolo and colleagues (2011) tried to assess implant osteointegration, as well as the course of bone regeneration and the healing process, following a sinus lift procedure using PRF as a filling material in association with Bio-Oss. They lifted the maxillary sinus of 23 patients to place implants into the posterior maxillary region. Before inserting the implant, they placed a small quantity of filling material in the cavity. For this purpose a bone fragment, stored in saline solution, was employed by being ground and then mixed with Bio-Oss and PRF. No pain to percussion, no sign of tissue damage in the soft peri-implant tissues, the presence of an optimal primary stability of the inserted implants, and a significant increase in the peri-implant bone density were reported in the patients (8). 

*3. Plastic Surgery*

Sclafani (2011) performed a cohort study to evaluate the clinical safety and efficacy of the use of autologous PRFM in facial plastic surgery. A total of 50 patients with at least 3 months of follow-up, who were treated by the author with PRFM for aesthetic purposes were reviewed for patient satisfaction, objective clinical results, and adverse events. Most patients were treated for deep nasolabial folds, while volume-depletion of the midface region, superficial rhytids, and acne scars were other commonly treated areas. No patients reported any swelling lasting longer than 5 days, and most noted only minimal bruising lasting for 1 to 3 days. Most patients were satisfied with the results of their treatments, although 1 patient felt that there was limited or no improvement after 2 treatments. He concluded that autologous PRFM treatment is a well-tolerated, excellent choice for use in the face (9). 

*4. Cartilage Reconstruction*

Haleem and colleagues (2010) completed a pilot study to test the hypothesis that platelet-rich fibrin glue (PR-FG) can be used clinically as a scaffold to deliver autologous culture-expanded bone marrow mesenchymal stem cells (BM-MSCs) for cartilage repair. They used autologous BM-MSCs that were culture expanded and placed on PR-FG intraoperatively. Then they transplanted the combined BM-MSCs and PR-FG into 5 full-thickness cartilage defects of the femoral condyles of 5 patients and covered it with an autologous periosteal flap. The patients were evaluated clinically at 6 and 12 months using the Lysholm and Revised Hospital for Special Surgery Knee (RHSSK) scores and radiographically by X-rays and magnetic resonance imaging (MRI) at the same time points. The tissue repair was rated in 2 patients arthroscopically after 12 months using the International Cartilage Repair Society (ICRS) Arthroscopic Score. The study reported that over a follow-up period of 12 months, all patients' symptoms improved. The average Lysholm and RHSSK scores for all patients showed statistically significant improvement at 6 and 12 months postoperatively (P<0.05). There was no statistically significant difference between the 6 and 12 months postoperative clinical scores (P= 0.18). ICRS arthroscopic scores were 8/12 and 11/12 (nearly normal) for the 2 patients who consented to arthroscopy. MRI of 3 patients at 12 months postoperatively revealed complete filling of the defect and complete surface congruity with native cartilage, whereas 2 patients showed incomplete congruity. They concluded that autologous BM-MSC transplantation on PR-FG as a cell scaffold may be an effective approach to promote the repair of articular cartilage defects of the knee in human patients (10).

*5. Bone Formation*

Liao and colleagues (2011) performed a study to examine whether a combination of autologous PR-FG with mesenchymal stem cells (MSCs) and MEDPOR used as guided tissue regeneration (GTR) could act as an osteogenic substitute and whether this treatment yields faster new bone formation than MEDPOR alone or PRFG plus MSC. They isolated MSCs from the bone marrow of dog ilium. They created a full thickness bony defect (1.5 × 1.5 cm) in the bilateral mandible angles of 24 dogs and divide them into 4 groups. In group I (n= 4), they used MEDPOR sheet as GTR and autologous PRFG/MSCs admixtures; in group II (n= 4) they used autologous PRFG/MSCs admixtures only; in group III (n= 4) they used MEDPOR sheet only as GTR; and group IV (n= 4) was used as a control with the defect left empty. The percentage of new bone regeneration was calculated by computerized tomography at 2 months and 4 months using Analyze version 7.0 software. The mandibles were harvested from all specimens at 4 months, and the grafted sites were evaluated by gross, histologic, and X-ray examination. Histologic examination revealed that the defect was repaired by typical bone tissue in groups I and II, whereas only minimal bone formation with fibrous connection was observed in the groups III and IV. In addition, muscle incarceration was found in groups II and IV without MEDPOR as GTR. Radiographic analysis at 16 weeks post-transplantation showed an average of 72.8 ± 8.02% new bone formation in group I, 53.3 ± 6.87% in group II, 26.6 ± 6.41% in group III, and 15.1 ± 2.37% in group IV. The researchers concluded that autologous PR-FG plus osteoinduced MSCs have good potential for bone regeneration. In combination with MEDPOR as GTR, bone regeneration is enhanced by preventing soft tissue ingrowth from hindering bone regeneration (11).

## Conclusion

The use of fibrin adhesives in many protocols has been well documented in the past 30 years (1, 2). Fibrin adhesives are often used in cardiothoracic and vascular surgery to seal diffuse microvascular bleeding (2), as well as to seal wound borders and facilitate cutaneous reuse in general and plastic surgery (3), and to reduce postoperative hematoma (4). PRF was first developed in France by Choukroun and colleagues (5). To produce PRF a centrifuged blood sample is used. PRF has been used to enhance the healing process in the patellar tendons of dogs and it was concluded that PRF increases the amount of tissue reconstruction (7). PRF has also been used in sinus lift procedures and demonstrated significant increase in peri-implant bone density and implant stability (8). In addition, PRF has been used effectively in plastic surgeries of the face and has been concluded to be an excellent choice for such surgeries (9). PRF could also facilitate repair of articular cartilage defects of the knee in combination with BM-MSC (10). PRF glue plus osteo-induced MSCs has also been used in bone regeneration (11). PRF is an effective biomaterial when used to enhance the post-surgical healing process and reduce the duration of the healing period, as has been shown in numerous studies. In this paper we report the details of a skin avulsion that was treated with PRF membrane.
